# Label-Free Quantification (LFQ) of Fecal Proteins for Potential Pregnancy Detection in Polar Bears

**DOI:** 10.3390/life12060796

**Published:** 2022-05-27

**Authors:** Erin Curry, Megan E. Philpott, Jessye Wojtusik, Wendy D. Haffey, Michael A. Wyder, Kenneth D. Greis, Terri L. Roth

**Affiliations:** 1 Center for Conservation and Research of Endangered Wildlife (CREW), Cincinnati Zoo & Botanical Garden, Cincinnati, OH 45220, USA; megan.philpott@cincinnatizoo.org (M.E.P.); jessye.wojtusik@omahazoo.com (J.W.); terri.roth@cincinnatizoo.org (T.L.R.); 2 Department of Reproductive Sciences, Omaha’s Henry Doorly Zoo and Aquarium, Omaha, NE 68107, USA; 3 UC Proteomics Laboratory, Department of Cancer Biology, University of Cincinnati College of Medicine, Cincinnati, OH 45267, USA; dominiwd@ucmail.uc.edu (W.D.H.); wyderma@ucmail.uc.edu (M.A.W.); greiskd@ucmail.uc.edu (K.D.G.)

**Keywords:** wildlife, non-invasive monitoring, pseudopregnancy, embryonic diapause, fecal proteomics

## Abstract

Reliable pregnancy diagnostics would be beneficial for monitoring polar bear (*Ursus maritimus*) populations both in situ and ex situ, but currently there is no method of non-invasive pregnancy detection in this species. Recent reports in several carnivore species described the identification of fecal proteins that may serve as pregnancy biomarkers; however, repeatability has been limited. The objective of the current analysis was to utilize an unbiased, antibody-free, label-free method for the identification and quantification of fecal proteins to determine if differences associated with pregnancy are detectable in polar bears. Protein was extracted from fecal samples (*n* = 48) obtained from parturient (*n* = 6) and non-parturient (*n* = 6) profiles each at four timepoints: pre-breeding season, embryonic diapause, early placental pregnancy, and mid-placental pregnancy. Protein was prepared and analyzed on the Thermo Orbitrap Eclipse nanoLC-MS/MS system. A total of 312 proteins was identified and quantified; however, coefficients of variation (CV) were high for both abundance ratio variability (384.8 ± 61.0% SEM) and within group variability (86.8 ± 1.5%). Results of this study suggest that the inconsistencies in specific protein concentrations revealed previously by antibody-based assays may not be due to that methodology’s limitations, but rather, are reflective of true variation that exists among samples.

## 1. Introduction

The ability to detect pregnancy in wildlife species holds value in animal conservation research and animal management, both in situ and ex situ. In field settings, the accurate assessment of pregnancy of individuals allows the evaluation of a population′s response to resource availability or environmental stressors and may aid in predicting population growth. These data can be used to inform wildlife managers and to determine the impact of anthropogenic factors that threaten a population’s health and subsistence. For animals in managed care, such as those in zoological settings, pregnancy determination may guide husbandry practices, ensuring that species-specific needs are provided to support offspring survival. There are several approaches to detecting pregnancy in wildlife species, including ultrasonography [1,2,3,4,5], analysis of urinary or fecal biomarkers such as progesterone [6,7], relaxin [8,9], or 13,14-dihydro-15-keto-PGF2a (PGFM) [10,11,12], and detection of hormones found in serum, such as pregnancy associated glycoproteins (PAGs) [13,14]. Nevertheless, the development of a reliable method to diagnose pregnancy has proven elusive in many species, despite this menu of diagnostic tools.

Polar bears (*Ursus maritimus*) exhibit an enigmatic complex of reproductive phenomena, including reproductive seasonality [15], presumed induced ovulation [16], embryonic diapause [17] resulting in a variable gestation length, and an apparently obligate pseudopregnancy in the absence of true pregnancy [18]. They breed in the springtime [18,19] and undergo delayed implantation until the embryo resumes growth in early autumn. True gestation, or placental pregnancy, is thought to last approximately 60 days [20]. In U.S. zoos, only ~10% of females produce cubs each year [20,21], even though nearly all are observed mating during breeding season. Neither the timing nor the cause of reproductive failure have been established, due largely to a deficiency of monitoring methods, including a lack of a proven non-invasive pregnancy test at any stage of gestation. Currently, polar bears are deemed pregnant or non-pregnant retrospectively, based on the occurrence of a parturition event.

The most commonly utilized reproductive monitoring techniques in wildlife focus on fecal steroid metabolite analyses [7] because the need for animal restraint, training, anesthesia, and/or significant deviations to daily husbandry routines are eliminated. In many species, the characterization of fecal progesterone metabolites allows tracking of ovarian cycles, with protracted luteal phases following ovulation and copulation suggestive of the pregnant state [22]. Although sustained elevated progesterone metabolite concentrations are useful in determining pregnancy status in many species, such as felids [22] and rhinoceros [23], in carnivores that exhibit embryonic diapause, such as polar bears, nearly all females exhibit increased progesterone concentrations following corpus luteum reactivation, regardless of the presence of an embryo [3,18,24,25]. The progesterone patterns associated with these pseudopregnancies are indistinguishable from those of pregnant females [24,26] in both magnitude and duration, [26,27]. Consequently, the utilization of progesterone concentrations for pregnancy diagnosis in species that experience both embryonic diapause and pseudopregnancy is largely a futile endeavor.

In pursuit of novel physiological biomarkers suitable for discerning the pregnant state, recent exploratory efforts have shifted attention from fecal steroid metabolites to fecal proteins. Several reports have described fecal protein candidates that may serve as pregnancy indicators in wildlife species, such as polar bears [28], cheetahs (*Acinonyx jubatus*) [29,30], and mink (*Neovison vison*) [31]; however, to date, none have been adapted into a bench side diagnostic assay. Original studies conducted by our lab [28,31] relied on two-dimensional differential gel electrophoresis (2D-DIGE) followed by mass spectrometry to identify proteins differing in abundance between pregnant and pseudopregnant females; however, replication of findings has been limited and attempts to develop enzyme-immunoassays (EIAs) to target individual proteins revealed wide fluctuations in intra-individual day-to-day excretion patterns [32], rendering them difficult to interpret. It is possible that limitations and/or biases inherent to the original discovery methods [33], such as dye biases associated with fluorescent [28,31] and isobaric [29] protein labeling, may preclude reproducibility with antibody-based techniques.

Furthermore, prior to collection, fecal samples utilized for these studies likely were subject to ambient temperatures, sunlight, and/or residual cleaning agents on habitat surfaces, all of which may contribute to protein denaturing within the sample [34,35,36,37]. In addition to incubation in the gastro-intestinal tract with digestive enzymes, it seems likely that fecal proteins are subject to some degree of degradation inherent to the sample matrix. Consequently, it is possible that antibody-based methods of protein detection, such as EIAs, are not suitable for quantifying fecal proteins which likely have altered antibody-binding sites, thus impeding accurate quantification by antibodies [37]. Therefore, we hypothesized that a protein quantification method that is unbiased, label-free, and not contingent on immunoassay-based quantification methods would allow detection and quantification of all proteins and peptides within a sample, and may illuminate differences associated with the pregnant state.

Label Free Quantification (LFQ) using mass spectrometry offers an untargeted, unbiased, label-free method of comparative profiling that does not utilize antibodies and has been shown to improve consistency and reproducibility of protein detection and quantification [38]. Despite growing in popularity for biomarker discovery in human clinical studies [39,40,41,42], the use of LFQ in fecal proteomics is limited mainly to the study of cancer and inflammatory bowel diseases [39,43]. There currently are no reports of the use of LFQ methodologies to investigate fecal proteins as biomarkers of any physiological condition in wildlife species. The objective of the current analysis was to utilize LFQ for the identification and quantification of protein components of polar bear fecal samples to determine if differences associated with pregnancy are detectable.

## 2. Materials and Methods

### 2.1. Animal Use Statement

Fecal samples were collected from zoo-housed polar bears as part of on-going reproductive monitoring studies conducted by scientists at the Center for Conservation and Research of Endangered Wildlife (CREW) at the Cincinnati Zoo & Botanical Garden (Cincinnati, OH, USA). All samples were collected non-invasively as part of routine husbandry practices.

### 2.2. Subjects and Sample Selection

All samples (*n* = 48) were collected non-invasively from polar bears (*n* = 10) living in North American zoological institutions (*n* = 8) from 2011–2016. Bears were born in a zoo (*n* = 6) or were wild orphans that had been brought into a zoo as cubs (*n* = 4) and ranged in age from 5.2 y to 15.9 y at the time of sample collection. Diets varied by institution, by season, and likely even within institution by day, but generally were composed of raw meat, fruits and vegetables, dry pellets formulated for feline or canine [44,45], and enrichment items, such as ox tail or live fish.

Samples were retrospectively selected from parturient (*n* = 6) and non-parturient (*n* = 6) profiles. Females were classified as parturient or non-parturient based on the production of cubs; two females served in both groups in different years of the study, resulting in a total of 12 profiles analyzed. Parturient females were housed with a male during breeding season, and half were housed with an additional female, whereas non-parturient females were not housed with an intact male, but had one-to-two conspecifics living at the same institution. Parturient females were primiparous or multiparous, whereas all non-parturient females were nulliparous at the time of sample collection. Animal management and husbandry were at the discretion of each institution; no changes were implemented for the purpose of this study.

Samples were selected from each profile (*n* = 12) at four different timepoints: pre-breeding season, embryonic diapause, early placental pregnancy, and mid-placental pregnancy. Pre-breeding season (Jan 26 ± 3.50 d) samples were collected prior to the first observed mating event between the female and a male. For non-parturient females, samples were date-matched to the parturient group. Because placental pregnancy in polar bears lasts ~60 days and is fixed in duration, it can be deduced that embryos are in diapause from the blastocyst stage (~7 days post-breeding) to ~60–70 days pre-partum, when embryonic growth resumes and placentation commences. Therefore, samples representing the embryonic diapause timepoint (Aug 1 ± 0.45 d; 127.3 ± 16.9 d pre-partum) were selected based both on season (subsequent to breeding season) and on mean parturition dates. Early placental pregnancy (Oct 21 ± 3.89 d; 44.8 ± 0.70 d pre-partum) and mid-placental pregnancy (Nov 7 ± 3.74 d; 30.8 ± 0.40 d pre-partum) sample dates were calculated based on parturition events. Because parturition did not occur in the non-parturient group, sample dates by calendar day were matched to the parturient group. Age of females (10.24 ± 0.48 y) did not differ between groups (Student’s *t*-test; *p* > 0.05). Additionally, samples were age-matched between groups to control for potential effects of storage time on protein degradation (Student’s *t*-test; *p* > 0.05).

Following excretion, fecal samples (~100 g) were picked up during normal husbandry practices, usually within 24 h of defecation, transferred into plastic bags labeled with the animal’s name/ID and the sample date and then stored at −20 °C until shipping to CREW, usually with six months of collection. Upon receipt, samples were thawed once, refrozen, and then stored until utilization in the current analysis.

### 2.3. Sample Preparation

Approximately 0.5 g of each frozen sample (*n* = 48) was weighed into a 15 mL conical tube and suspended in 5.0 mL phosphate buffered saline (PBS) containing protease inhibitor cocktail (Roche Diagnostics GmbH, Mannheim, Germany). Sample processing and analysis were performed at the University of Cincinnati Proteomics Laboratory (Cincinnati, OH, USA). After intermittent vortexing and rotating for 60 min, bulk particulates were removed by centrifugation at 4 °C and 5000× *g* in a refrigerated swinging bucket rotor. The resulting clarified supernatant (3 mL) was subsequently filtered through a 0.45 µm filter. A Pierce™ 660 nm Protein Assay Kit (ThermoFisher Scientific, Florence, KY, USA) was used to quantify protein concentration in each sample and then 500 ng from each was prepared for SDS-PAGE with silver staining to assess protein separation and banding patterns. Then, 25 µg of each sample in gel solubilization buffer was heated to 100 °C for 10 min and loaded on a 1.5 mm, 4–12% Bis-Tris gradient SDS-PAGE gel (Invitrogen, Waltham, MA, USA) using 3-(N-morpholino) propane sulfonic acid (MOPS) buffer and electrophoresed at 125 V for 15 min until the samples ran ~2 cm into the gel. Pre-stained molecular weight protein markers were used adjacent to each sample to define the gel region containing the total proteins. Gel sections containing proteins were excised, reduced with dithiothreitol, alkylated with iodoacetamide, digested overnight with trypsin, and recovered, all as described previously [46]. The extracted peptides were dried, reconstituted in 0.1% formic acid in preparation for mass spectrometry analysis.

### 2.4. LFQ Mass Spectrometry Workflow

Label Free Quantitation (LFQ) mass spectrometry data were collected on an Orbitrap Eclipse mass spectrometer coupled to a Dionex Ultimate 3000 RSLCnano system (ThermoFisher Scientific, Florence, KY, USA). Samples were injected onto a 5 mm nanoviper μ-Precolumn (i.d. 300 μm, C18 PepMap 100, 5.0 μm, 100 Å) from ThermoFisher Scientific at 5 µL/min in formic acid/H_2_O 0.1/99.9 (*v*/*v*) for 5 min to desalt and concentrate the samples. For the chromatographic separation of peptides, the trap-column was switched to align with the EASY-Spray column PepMap RSLC C18 with a 150 mm column (i.d. 75 μm, C18, 3.0 μm, 100 Å). The peptides were eluted using a variable mobile phase (MP) gradient from 98% phase A (formic acid/H2O 0.1/99.9, *v*/*v*) to 32% phase B (formic acid/acetonitrile 0.1/99.9, *v*/*v*) for 90 min at 300 nL/min. MS1 data were collected in the Orbitrap (120,000 resolution; maximum injection time 50 ms; AGC 4 × 10^5^). Charge states between 2 and 6 were required for MS2 analysis, and a 20 s dynamic exclusion window was used. Cycle time was set at 2.5 s. MS2 scans were performed in the ion trap with HCD fragmentation (isolation window 0.8 Da; NCE 30%; maximum injection time 40 ms; AGC 5 × 10^4^). The data were recorded using Xcalibur 4.3 software (ThermoFisher Scientific, Florence, KY, USA).

Protein identification and LFQ were achieved using Proteome Discoverer 2.4 (ThermoFisher Scientific, Florence, KY, USA) searched against the Uniprot database of all combined *Ursus* species proteins with the Sequest HT search algorithm and using a modified LFQ standard processing and consensus workflows. The processing workflow included the mass recalibration node (spectrum files RC) along with the standard spectrum selector, Minora Feature Detector, Sequest HT and Percolator nodes. The precursor detector node was used to help minimize chimeric spectra. The precursor mass tolerance was 10 ppm and the fragment mass tolerance was set to 0.02 Da, with two missed trypsin cleavages and variable peptide modifications including oxidized methionine, and carbamidomethyl cysteine, and N-terminal protein modifications of Acetyl, Met loss. False discovery rate (FDR) tolerances in the Percolator node were set to 0.01 for high confidence and 0.05 for medium confidence. Protein abundance calculations across the full data set were based on summed abundance of extracted ion profiles from the collective MS1 profiles of peptides unique to the identified protein with ratios calculated using pairwise comparisons. The maximum allowed fold-change was 100.

### 2.5. Data Analysis

Data analysis included total proteins detected and quantified, and the abundance ratio of protein changes across the parturient/non-parturient timepoints. Spearman correlation was used to evaluate reproducibility and principal component analysis (PCA) was used to assess clustering within group. Regression analyses were performed in R ver. 4.1.0 (R Core Team 2021). Models were created with the *lme4* package, *p*-values were generated with the *lmerTest* package using Satterthwaite’s degrees of freedom method, effect sizes were calculated with the *emmeans* package, and graphics were created with the *ggplot2* package [47,48,49,50]. Abundance data for all proteins identified with two or more peptides were log_2_-transformed before analysis, and any proteins with missing values were dropped from the dataset. A linear mixed-effects model of abundance data was constructed with time-point and pregnancy status included as fixed effects with an interaction and animal ID included as random effects. Post-hoc comparisons were made using estimated marginal means with a Tukey adjustment for multiple comparisons

Differential expression was estimated in R using the *limma* package [51]. Raw abundance data for each individual in the study was included for all proteins identified with two or more peptides with scale normalization applied, and missing values were assigned 0. Linear models were fitted for each protein in the analysis using the weighted least squares method. Comparisons were made between parturient and non-parturient samples at corresponding timepoints, and the interaction between pregnancy status and timepoint. Significance was determined using the Benjamini-Hochberg false discovery rate adjusted *p*-value.

To categorically assess the proportion of the presence or absence of specific proteins, proteins identified with two or more peptides were categorized according to presence/absence at each condition (parturient or non-parturient) and each timepoint. Pearson’s chi-squared test was used to determine if there were significant differences in the presence/absence of each protein between groups at each timepoint. The Benjamini-Hochberg false discovery rate also was used to adjust *p*-value for this analysis.

To allow inferences to be deduced by grouping proteins into functional categories, gene ontology (GO) analyses were performed [52]. Proteins identified with a ≥2-fold change between groups at different timepoints were used to analyze distribution of GO terms. GO terms were extracted for proteins upregulated in parturient or non-parturient samples at each timepoint, and the distribution of GO terms by molecular function, cellular component, and biological process was quantified by searching accessions on Uniprot [52].

## 3. Results

### 3.1. Protein Extraction and Abundance

**Figure 1:**
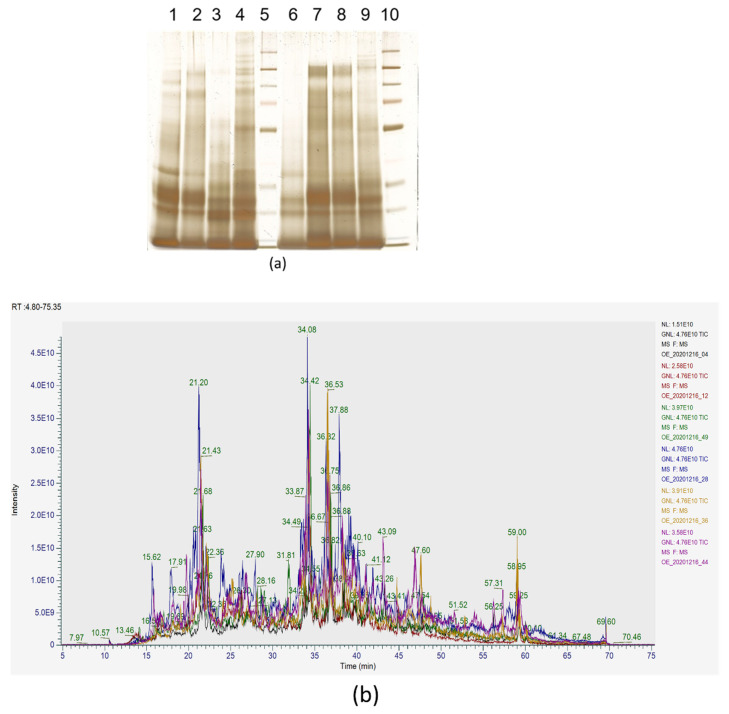
Images depicting protein abundance among samples: (**a**) Representative image of a silver-stained gel containing eight protein samples extracted from polar bear feces. The lack of distinct protein banding and overall smeared appearance is indicative of protein degradation. Lanes 1–4 contain samples from a parturient female, collected at pre-estrus, diapause, early placental pregnancy, and mid-placental pregnancy. Lanes 6–9 contain samples of corresponding timepoints from a non-parturient female. Lanes 5 and 10 are molecular weight ladders; (**b**) Figure 1b is a representative Total Ion Chromatogram (TIC) showing early pregnancy profiles from the LC-MS analysis of 2.5 µg of trypsin digested samples, with consistent levels of peptides in each sample for comparative analysis. Note that the intensity units are scientific notation where 5.0E9 represents 5 × 10^9^.

Protein assays indicated at least 150 µg of protein were available for each sample. Silver-stained gels containing 500 ng of each protein sample showed a greater abundance of low molecular weight proteins, indicative of general protein degradation (Figure 1a). An overlay of the Total Ion Chromatogram (TIC) profiles from the LC_MS analysis showed consistent total signals across the sample set indicating near equal amounts of protein digestion loaded for each injection (Figure 1b).

**Figure 2:**
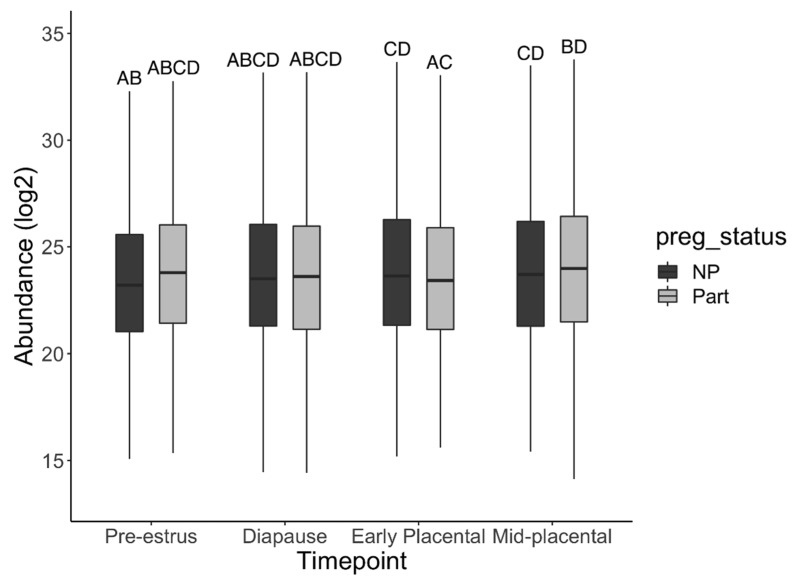
Estimated marginal means for the total protein abundance at each pregnancy status by timepoint. Bars represent 95% confidence interval. Bars sharing the same letters are not significantly different.

Overall protein abundance did not differ significantly between parturient and non-parturient groups in the model (*p* = 0.12). However, total protein abundance in pre-estrus and diapause timepoints differed significantly from mid-placental (*p <* 0.01 and *p <* 0.05, respectively; Figure 2). Non-parturient samples had a significantly lower abundance in pre-estrus than in early (*p <* 0.01) or mid-placental pregnancy (*p <* 0.01; Figure 2). Parturient samples had a significantly lower abundance in early compared to mid-placental pregnancy (*p <* 0.01).

### 3.2. Protein Identification and Quantification

A total of 312 proteins with a minimum of two peptides was identified and quantified. The most abundant proteins are listed in Table 1, which are predominantly involved in digestive processes and immune function. See Supplemental File S1 for list of all proteins detected. Of the proteins identified, 191 (61.2%) were detected across all samples and 123 showed >2-fold change in the mean protein abundance ratio between parturient and non-parturient groups for at least one timepoint; however, CVs were high for both abundance ratio variability (384.8 ± 61.0% SEM) and within group variability (86.8 ± 1.5%) by timepoint, indicating low reproducibility and inconsistency of individual protein abundances in grouped samples.

**Figure 3:**
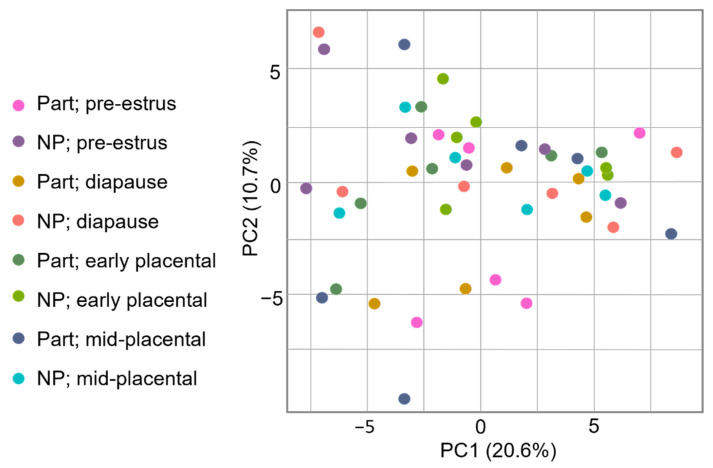
Principal component analysis (PCA). No clustering by group by timepoint is observed.

**Figure 4:**
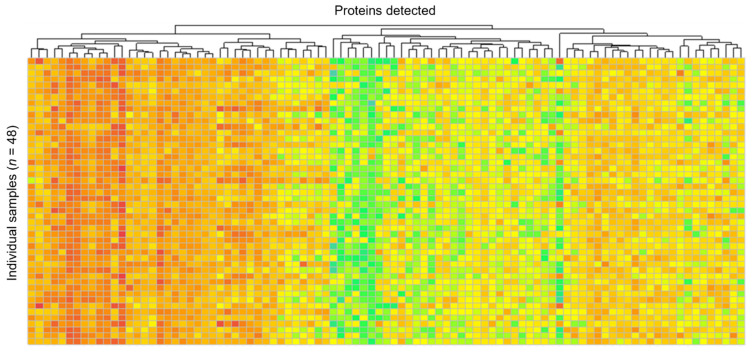
Heatmap showing relative expression of proteins detected (*X*-axis) by individual sample (*Y*-axis). Little to no banding by group was detected.

The Spearman correlation, which evaluates the pairwise reproducibility for all biological replicates in a group, was low for all comparisons with a mean of 0.362, indicating biological variability within samples collected at the same timepoint within the group. Likewise, PCA provides a measure of how similar divergences within a group can result in the clustering of samples by group; however, PCs 1 and 2 only explained 30.7% of the variation, and consequently PCA showed little or no clustering of samples by groups, indicating low reproducibility and inconsistency in grouped samples (Figure 3). Lastly, a heatmap showed no defined banding by group and significant variability across the dataset (Figure 4).

**Figure 5:**
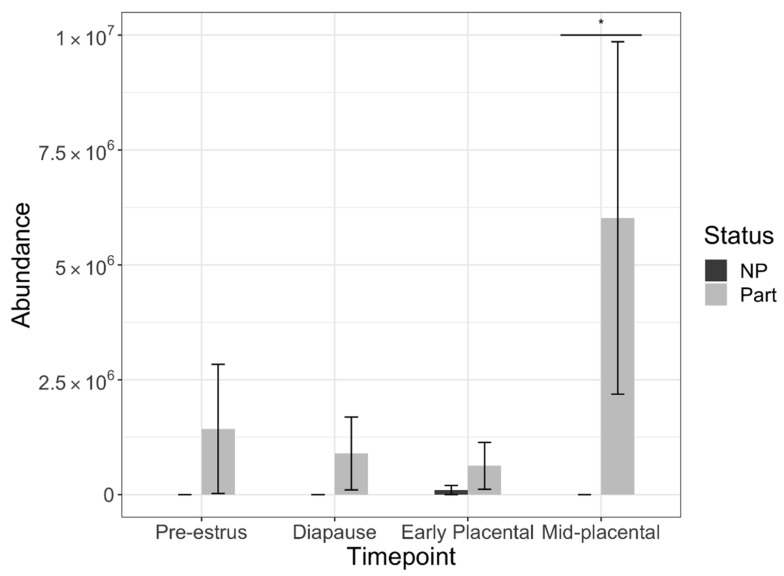
Bar graph showing mean abundance of eosinophil peroxidase by pregnancy status (non-parturient (NP) and parturient (Part)). Error bars are standard error of the mean. Asterisk (*) indicates significant difference within timepoint.

One protein, eosinophil peroxidase (accession: A0A452SXQ5) was significantly higher between parturient and non-parturient groups (*p >* 0.05) and, when analyzed using the interaction of pregnancy status and timepoint, was found to be significantly higher during mid-placental pregnancy (Figure 5; adj. *p =* 0.008, log2 fold change = −18.19); however, it was not significantly higher within group when compared to the other timepoints within group (*p >* 0.05).

When presence versus absence of individual proteins was assessed, no proteins were both present in all samples in one group and absent in all samples of the other group at the same timepoint; however, seven proteins were identified as being present in most samples per group (at least four of six samples; 66.7%) and absent in all samples from the other group at the corresponding timepoint. During pre-estrus, Ig-like domain-containing protein (A0A452R730) was present in 66.7% samples of the non-parturient group but was absent in all samples in the parturient group. During early placental pregnancy, MHC_II_beta domain-containing protein (A0A452QMZ0) and aspartyl aminopeptidase (A0A384C1U0) were present in 66.7% and 83.3% of parturient samples, respectively, but were not detected in non-parturient samples. During early placental pregnancy, low-quality protein titin (A0A384BT37) was present in 83.3% of samples in the non-parturient group but was not detected in the parturient group at the same timepoint. During mid-placental pregnancy, adiponectin (A0A452TBN0), collagen alpha-6(IV) chain (A0A3Q7U2T4), and eosinophil peroxidase (A0A452SXQ5) were present in 66.7, 66.7, and 83.3% of parturient samples, respectively, but were absent in non-parturient samples collected at the same timepoint.

### 3.3. Gene Ontology

Analysis of GO terms was performed for proteins with a 2-fold change or greater in parturient or non-parturient samples at each timepoint to allow inferences to be deduced by grouping proteins into functional categories. The complete distribution of GO terms by molecular function, cellular component, and biological process are included in Supplemental File S2. Proteins with at least a 2-fold change showed enriched GO terms for cellular processes, metabolic processes, cellular anatomical entity, binding, and catalytic activity. Differences between parturient and non-parturient were observed primarily in mid-placental pregnancy in the following terms: binding, cellular process, biological regulation, response to stimulus, localization, biological process involved in interspecies interaction between organisms, and immune system process.

## 4. Discussion

Results of the LFQ revealed that there is significant variation in specific protein concentrations by sample that are not necessarily reflective of pregnancy status. Findings suggest that the variabilities in concentrations of specific proteins reported previously from antibody-based assays may not be due to that methodology’s limitations but, rather, are reflective of the true variation that exists among samples. In the current study, the CVs were high for both abundance ratio variability and within group variability by timepoint, indicating low replicability and high variation of individual protein abundances in samples grouped by pregnancy status and timepoint. It is likely that uncontrolled study cohorts, diverse diets, and inconsistent gut passage time all contributed to the variability in protein abundances observed, potentially concealing any differences due to the pregnant state.

Although no single proteins emerged as a pregnancy-specific biomarker, several were more likely to be detected in either parturient or non-parturient samples at matched timepoints. During pre-estrus, Ig-like domain-containing protein was higher in the non-parturient group compared to the parturient group. During early placental pregnancy, both MHC_II_beta domain-containing protein and aspartyl aminopeptidase were detected in parturient samples but were not found in non-parturient samples, whereas titin was found in most of the non-parturient samples, but none of parturient samples. During mid-placental pregnancy, adiponectin, collagen alpha-6 (IV) chain, and eosinophil peroxidase were present in most parturient samples but were absent in non-parturient samples. With the exception of eosinophil peroxidase, none of these differences was statistically significant, either by differential expression analyses or Pearson chi-square test, and eosinophil peroxidase was not significantly higher in mid-placental pregnancy when compared to the other timepoints within the parturient group.

Arguments could be constructed for all of these proteins and their prospective roles in fertility, pregnancy establishment, fetal growth, and/or pregnancy maintenance; however, it is worth noting that no protein identified in the current study was detected as a candidate in previous studies evaluating differences in fecal proteins in pregnant versus non-pregnant carnivores [28,29,31,53]. Overall, consensus has been scant regarding which proteins transpire as pregnancy protein biomarkers among these efforts. Considering the relatively small number of biological replicates utilized per study and the ample number of total proteins discovered per analysis, it warrants consideration that the differences reported are only artifacts due to small sample size and high variability. Despite the discrepancies, all studies consistently point to proteins related to digestion, immune function, or tissue remodeling. It is possible that the differences observed are simply reflective of the protein population in fecal samples, or they may be due to unique nutrient absorption needs during pregnancy, regulation of the immune milieu, or modulation of placental or vascular remodeling [53] that occur during pregnancy.

There is growing interest in proteomic analyses and biomarker discovery in wildlife species and non-model organisms [54,55], especially relating to improved understanding of unique adaptations and how they might provide insight into human disease, such as cancer resistance [56,57,58], metabolism [59,60], and organ regeneration [61,62]. There is boundless potential for discovery in the proteomes of exotic species, not only for human health, but also for developing improved monitoring systems for wildlife species themselves. Proteomic research is largely driven by genomics, and, to date, the genomic annotation of non-model organisms has lagged significantly behind traditional model organisms. Fortunately, the cost of genome assembly is decreasing [63], so the number of completed annotated genomes will continue to increase. If a genome of a particular species is unavailable, homology searching of the database of a closely related species can be employed [54]. In the current study, the databases of other bear species, including brown bears (*Ursus arctos*) were queried. Brown bears are likely a reliable proxy for polar bears, as the hybridization of the two species has been well documented [64], indicating that they possess a high degree of protein homology.

Ideally, future attempts at fecal protein biomarker detection in wildlife should focus on populations where variables such as age, diet, housing conditions, and sample collection can be better controlled among individuals. As more animals are trained for voluntary blood collection in zoological settings, it is possible that serum may become the preferred biological matrix for biomarker discovery studies, as recent reports indicate its usefulness in monitoring the reproductive health of wild bear populations [65]. Results of this study underscore the challenges of fecal protein analyses in wildlife, especially when samples are collected opportunistically from heterogenous populations.

## Figures and Tables

**Table 1:** Most abundant fecal proteins identified.

UniProtID	Protein	Mean Raw Abundance
A0A452SML2	Carboxypeptidase A1	14,329,861,669
A0A452SXJ4	Uncharacterized protein	13,577,232,635
A0A452UQI9	Ig-like domain-containing protein	6,370,583,108
A0A452UQ85	IGv domain-containing protein	6,192,069,758
A0A3Q7UBB8	IgGFc-binding protein	5,536,351,190
A0A3Q7W0E2	Titin isoform X2	4,528,398,405
A0A3Q7VT65	Alkaline phosphatase	2,394,052,435
A0A384BSZ9	Dipeptidyl peptidase 4	2,189,880,724
A0A452U793	Peptidase S1 domain-containing protein	2,139,152,673
A0A452QAI0	Ig-like domain-containing protein	1,425,672,788

## Data Availability

Not applicable.

## Supplementary Materials

The following supporting information can be downloaded at: https://www.mdpi.com/article/10.3390/life12060796/s1. File S1: Proteins with two or more peptides identified. File S2: Gene ontology (GO) enrichment graphs.
