# Supplementary figures and images for: Label-Free Quantification (LFQ) of Fecal Proteins for Potential Pregnancy Detection in Polar Bears
Source: Life (Basel). 2022 May 27;12(6):796. doi: 10.3390/life12060796 (PMC9225558; doi:10.3390/life12060796)

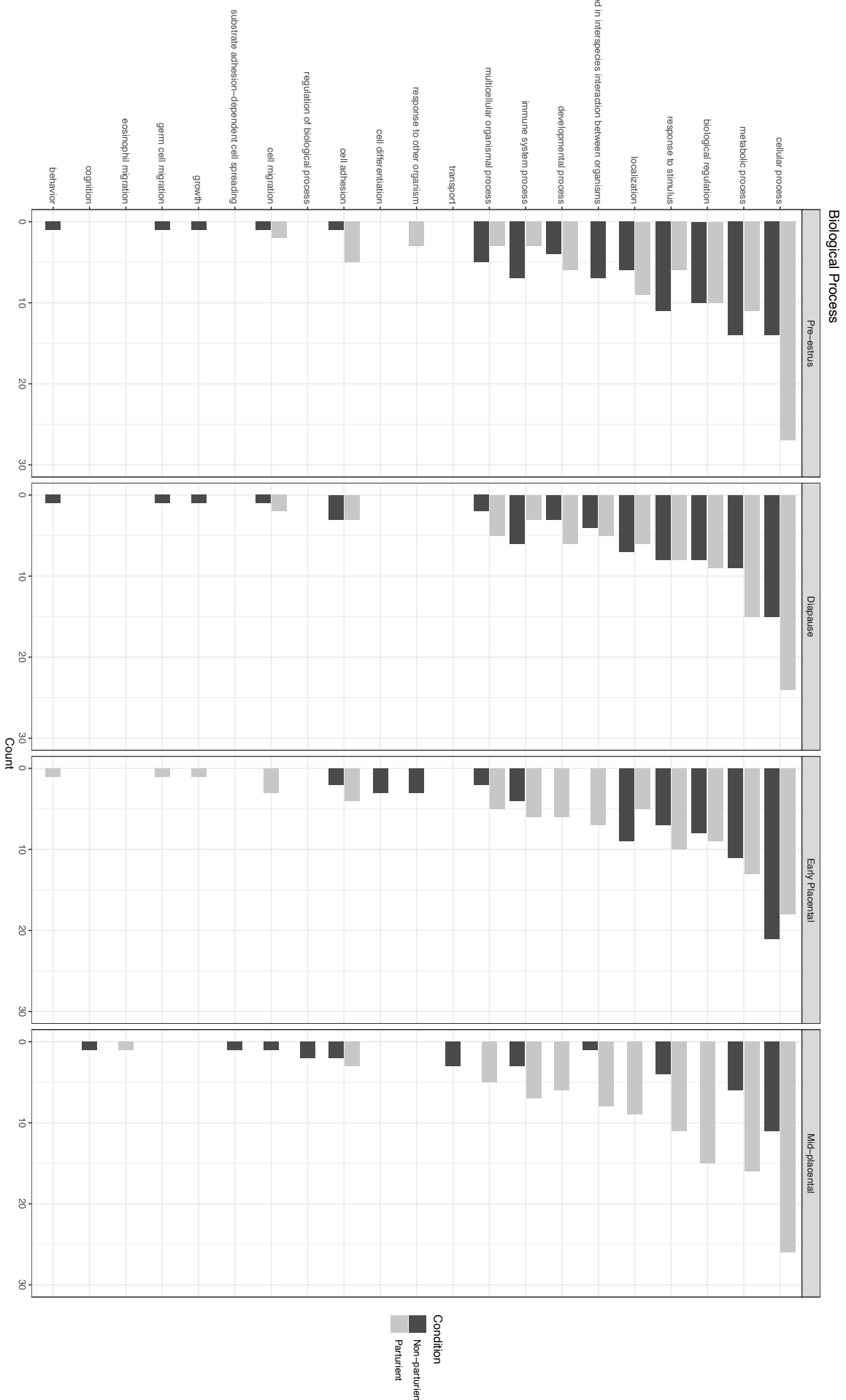

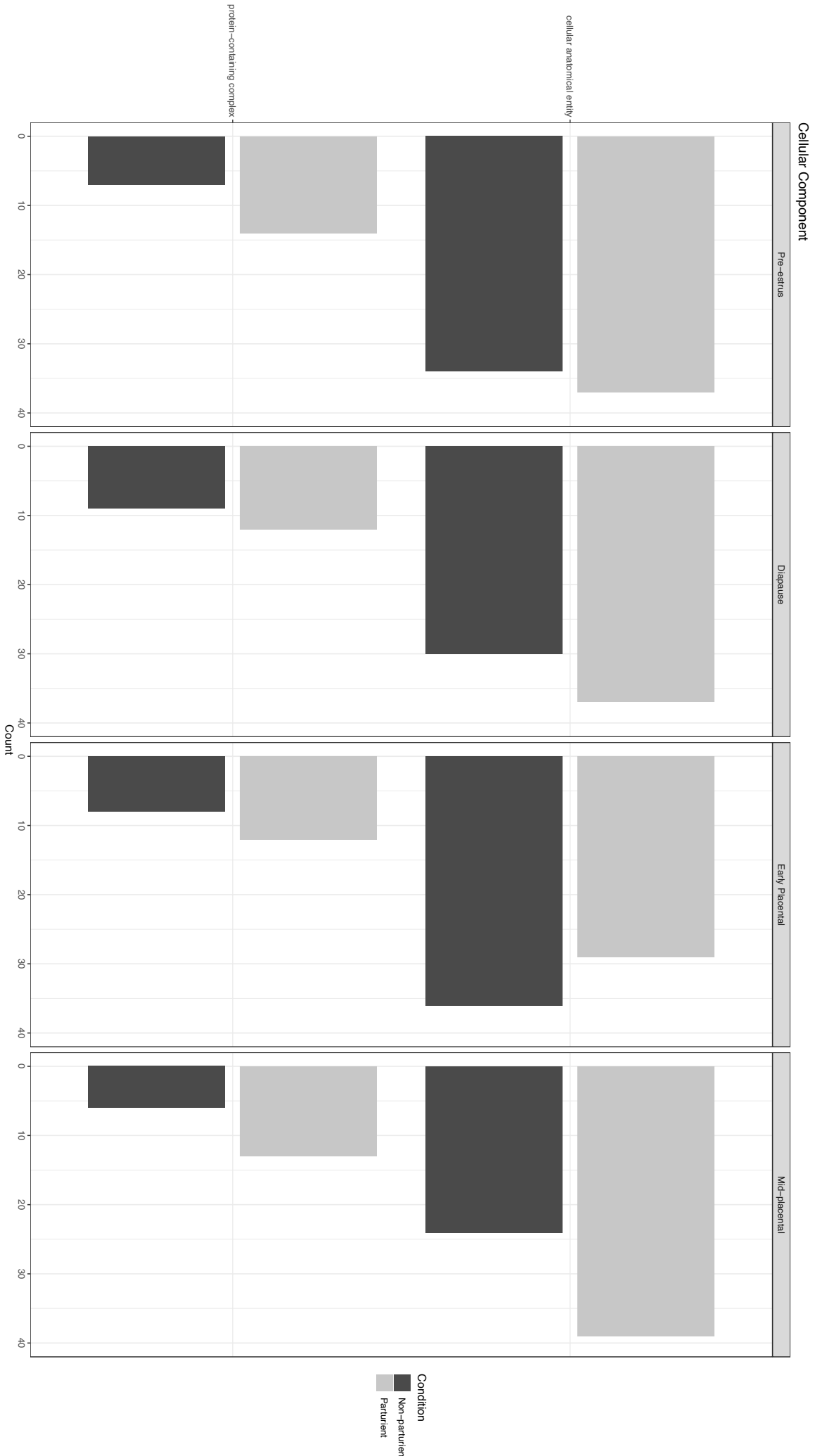

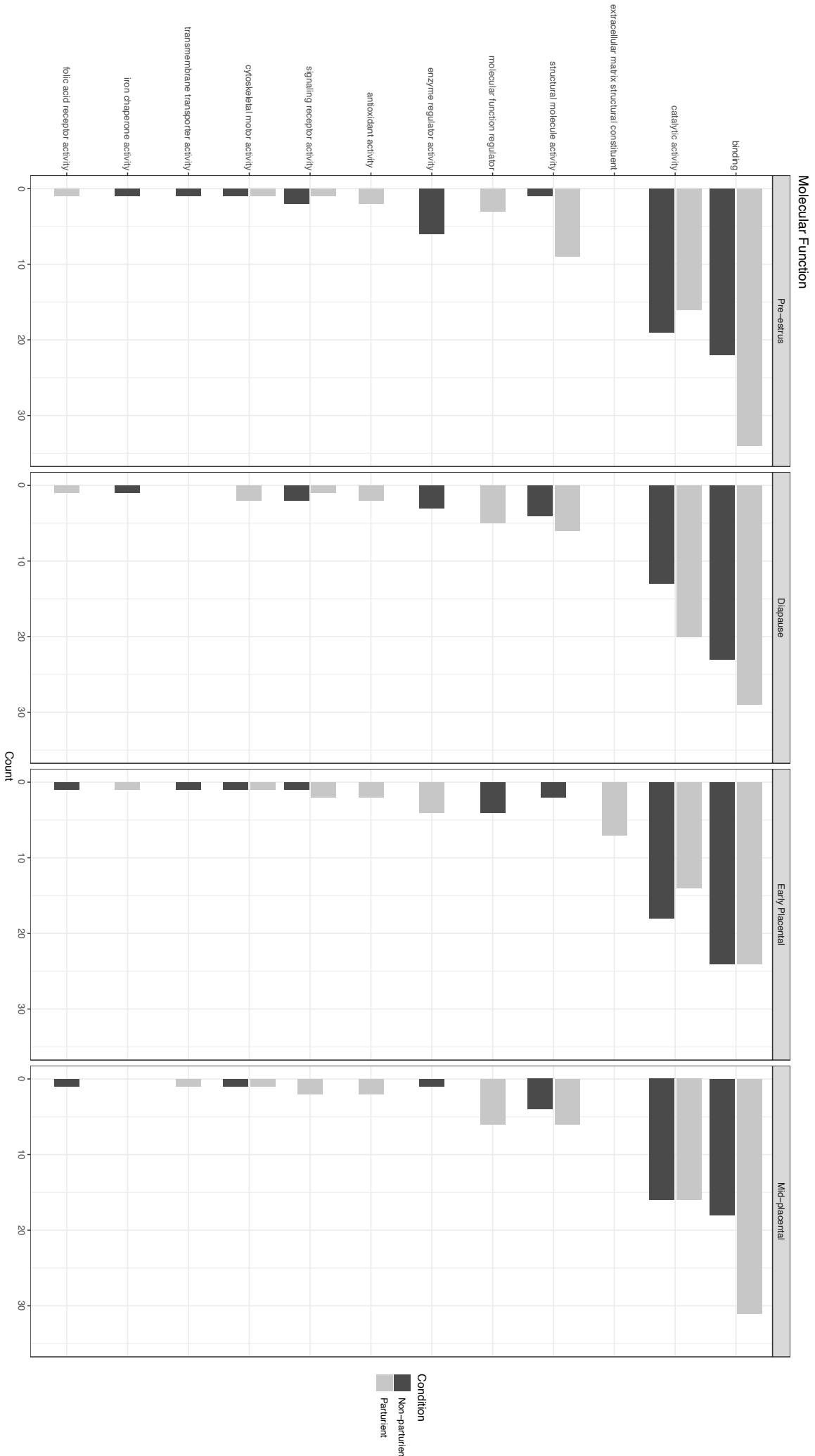

Supplement: Supplementary file 1 [file life-12-00796-s001.zip › Supplementary file 2_ GO terms.pdf]
